# (1,10-Phenanthroline-κ^2^ *N*,*N*′)bis­(2-thioxo-1,2-dihydro­pyridine-3-car­box­yl­ato-κ^2^ *O*,*S*)manganese(II)

**DOI:** 10.1107/S160053681001514X

**Published:** 2010-05-15

**Authors:** Wei-Qi Li

**Affiliations:** a Jinhua Radio and Television University, Jinhua, Zhejiang 321022, People’s Republic of China

## Abstract

In the title complex, [Mn(C_6_H_4_NO_2_S)_2_(C_12_H_8_N_2_)] or [Mn*L*_2_(phen)] (*L* = 2-mercaptonicotinate and phen = 1,10-phenanthroline), the central Mn^II^ atom is coordinated by two carboxylic O atoms and two thiol­ate S atoms of two *L* ligands and two N atoms from one phen mol­ecule, giving a distorted octa­hedral geometry. The pyridyl H atoms form strong N—H⋯O hydrogen bonds with the carbonyl O atoms of the adjacent mol­ecules, generating a chain structure propagating in [100].

## Related literature

For solvothermal synthesis with compounds containing carboxylate ligand systems see: Bröll *et al.* (1999 ▶). For the different structural forms and potential multiple bidentate coordinate possibilities of the H_2_ *L* ligand, see: Ma *et al.* (2003 ▶); Saleh *et al.* (1996 ▶); Zachariadis *et al.* (2003 ▶).
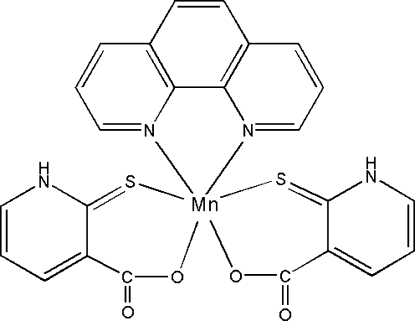


## Experimental

### 

#### Crystal data

[Mn(C_6_H_4_NO_2_S)_2_(C_12_H_8_N_2_)]
*M* *_r_* = 543.47
Triclinic, 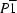

*a* = 7.2369 (7) Å
*b* = 11.0966 (12) Å
*c* = 15.1290 (17) Å
α = 105.177 (6)°
β = 90.079 (5)°
γ = 102.429 (5)°
*V* = 1142.9 (2) Å^3^
*Z* = 2
Mo *K*α radiation
μ = 0.80 mm^−1^
*T* = 296 K
0.43 × 0.09 × 0.07 mm

#### Data collection

Bruker APEXII area-detector diffractometer
Absorption correction: multi-scan (*SADABS*; Sheldrick, 1996 ▶) *T*_min_ = 0.918, *T*_max_ = 0.946
16022 measured reflections
4600 independent reflections
3945 reflections with *I* > 2σ(*I*)
*R*_int_ = 0.020

#### Refinement

*R*[*F* ^2^ > 2σ(*F* ^2^)] = 0.031
*wR*(*F* ^2^) = 0.093
*S* = 1.06
4600 reflections
316 parameters
H-atom parameters constrained
Δρ_max_ = 0.50 e Å^−3^
Δρ_min_ = −0.45 e Å^−3^

### 

Data collection: *APEX2* (Bruker, 2006 ▶); cell refinement: *SAINT* (Bruker, 2006 ▶); data reduction: *SAINT*; program(s) used to solve structure: *SHELXS97* (Sheldrick, 2008 ▶); program(s) used to refine structure: *SHELXL97* (Sheldrick, 2008 ▶); molecular graphics: *SHELXTL* (Sheldrick, 2008 ▶); software used to prepare material for publication: *SHELXTL*.

## Supplementary Material

Crystal structure: contains datablocks I, global. DOI: 10.1107/S160053681001514X/pv2275sup1.cif

Structure factors: contains datablocks I. DOI: 10.1107/S160053681001514X/pv2275Isup2.hkl

Additional supplementary materials:  crystallographic information; 3D view; checkCIF report

## Figures and Tables

**Table 1:** Hydrogen-bond geometry (Å, °)

*D*—H⋯*A*	*D*—H	H⋯*A*	*D*⋯*A*	*D*—H⋯*A*
N1—H1*A*⋯O2^i^	0.86	1.86	2.654 (2)	152
N2—H2*A*⋯O4^ii^	0.86	2.00	2.7476 (19)	145

Symmetry codes: (i) 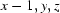; (ii) 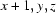.

**Fig. 1.**
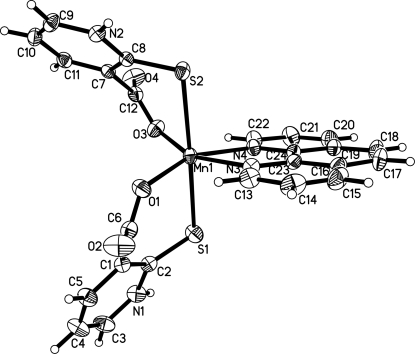
Perspective view of the structure of (I), showing the atom-numbering scheme. Displacement ellipsoids are drawn at the 30% probability level.

**Fig. 2.**
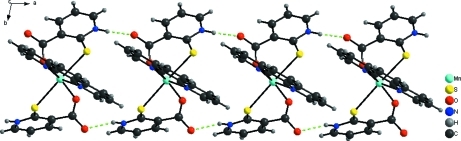
A view of the one-dimensional chain structure of (I). The N—H···O interactions are depicted by dashed lines.

## supplementary crystallographic information

### Comment

In contrast to a great deal of work on solvothermal synthesis with carboxylate ligands (Bröll *et al.*, 1999), there have been few reports of studies on mixed thiolato-pyridinecarboxylic ligands. 2-Mercaptonicotinic acid (H_2_L) is a multifunctional ligand containing one carboxyl group, one thiol group and a pyridyl N donor atom. In addition, as an equilibrium mixture of thiol and thione forms in solution, H_2_L is an interesting ligand in the thiolatopyridine system because of its different structural forms and potential multiple bidentate coordinate possibilities (Ma *et al.*, 2003; Saleh *et al.*, 1996; Zachariadis *et al.*; 2003). Herein in this article, the synthesis and structure of a new compound, [MnL_2_(phen)], is reported.

A perspective view of the title complex (I) is presented in Fig. 1. The central Mn^II^ atom is six-coordinated by two carboxylic O atoms of two L^2-^ ligands [Mn—O 2.0868 (14) and 2.0975 (13) Å], two thiolato S atoms of two L^2-^ ligands [Mn—S 2.6067 (6) and 2.6347 (5) Å] and two N atoms from one phen [Mn—N 2.2627 (15) and 2.2822 (15) Å], giving a distorted octahedral geometry. The adjacent molecules are linked by N—H···O intermolecular hydrogen bonds to form a one dimensional chain structure (Fig. 2).

### Experimental

A mixture of H_2_L (0.155 g, 1.0 mmol), MnCl_2_.4H_2_O (0.198 g, 1.0 mmol), phen (0.099 g, 0.5 mmol), and Na_2_CO_3_ (0.053 g, 0.5 mmol) in C_2_H_5_OH (2 ml)/H_2_O (16 ml) was placed in a Teflon-lined stainless steel vessel and heated at 433 K for 72 h, and then cooled to room temperature over 3 days. The resulting red crystals suitable for X-ray analysis were obtained in 30% yield.

### Refinement

The H-atoms were positioned geometrically and included in the refinement using a riding model with N—H = 0.86 Å and C—H = 0.93 Å and U_iso_(H) = 1.2U_eq_(N/C).

### Crystal data

**Table:**

[Mn(C_6_H_4_NO_2_S)_2_(C_12_H_8_N_2_)]	*Z* = 2
*M**_r_* = 543.47	*F*(000) = 554
Triclinic, *P*1	*D*_x_ = 1.579 Mg m^−^^3^
Hall symbol: -P 1	Mo *K*α radiation, λ = 0.71073 Å
*a* = 7.2369 (7) Å	Cell parameters from 7683 reflections
*b* = 11.0966 (12) Å	θ = 2.0–26.5°
*c* = 15.1290 (17) Å	µ = 0.80 mm^−^^1^
α = 105.177 (6)°	*T* = 296 K
β = 90.079 (5)°	Needle, red
γ = 102.429 (5)°	0.43 × 0.09 × 0.07 mm
*V* = 1142.9 (2) Å^3^	

### Data collection

**Table:**

Bruker APEXII area-detector diffractometer	4600 independent reflections
Radiation source: fine-focus sealed tube	3945 reflections with *I* > 2σ(*I*)
graphite	*R*_int_ = 0.020
ω scans	θ_max_ = 26.5°, θ_min_ = 2.0°
Absorption correction: multi-scan (*SADABS*; Sheldrick, 1996)	*h* = −9→9
*T*_min_ = 0.918, *T*_max_ = 0.946	*k* = −13→13
16022 measured reflections	*l* = −15→18

### Refinement

**Table:**

Refinement on *F*^2^	Primary atom site location: structure-invariant direct methods
Least-squares matrix: full	Secondary atom site location: difference Fourier map
*R*[*F*^2^ > 2σ(*F*^2^)] = 0.031	Hydrogen site location: inferred from neighbouring sites
*wR*(*F*^2^) = 0.093	H-atom parameters constrained
*S* = 1.06	*w* = 1/[σ^2^(*F*_o_^2^) + (0.0545*P*)^2^ + 0.2746*P*] where *P* = (*F*_o_^2^ + 2*F*_c_^2^)/3
4600 reflections	(Δ/σ)_max_ = 0.001
316 parameters	Δρ_max_ = 0.50 e Å^−^^3^
0 restraints	Δρ_min_ = −0.45 e Å^−^^3^

### Special details

**Table:**

Geometry. All esds (except the esd in the dihedral angle between two l.s. planes) are estimated using the full covariance matrix. The cell esds are taken into account individually in the estimation of esds in distances, angles and torsion angles; correlations between esds in cell parameters are only used when they are defined by crystal symmetry. An approximate (isotropic) treatment of cell esds is used for estimating esds involving l.s. planes.
Refinement. Refinement of *F*^2^ against ALL reflections. The weighted *R*-factor wR and goodness of fit *S* are based on *F*^2^, conventional *R*-factors *R* are based on *F*, with *F* set to zero for negative *F*^2^. The threshold expression of *F*^2^ > σ(*F*^2^) is used only for calculating *R*-factors(gt) etc. and is not relevant to the choice of reflections for refinement. *R*-factors based on *F*^2^ are statistically about twice as large as those based on *F*, and *R*- factors based on ALL data will be even larger.

### Fractional atomic coordinates and isotropic or equivalent isotropic displacement parameters (Å^2^)

**Table:**

	*x*	*y*	*z*	*U*_iso_*/*U*_eq_	
Mn1	0.19275 (4)	0.18006 (3)	0.772122 (17)	0.03375 (10)	
S1	0.00472 (7)	0.35983 (5)	0.78952 (4)	0.04714 (14)	
S2	0.36963 (7)	−0.00932 (5)	0.74720 (3)	0.04092 (13)	
O1	0.3743 (2)	0.28650 (14)	0.69924 (10)	0.0498 (4)	
O2	0.5749 (2)	0.46218 (16)	0.68907 (15)	0.0721 (5)	
O3	−0.01444 (18)	0.04438 (13)	0.67939 (9)	0.0439 (3)	
O4	−0.17076 (19)	−0.15020 (15)	0.60409 (12)	0.0582 (4)	
N1	−0.0514 (2)	0.49770 (16)	0.68254 (12)	0.0444 (4)	
H1A	−0.1632	0.4842	0.7025	0.053*	
N2	0.4711 (2)	−0.11197 (14)	0.58227 (10)	0.0343 (3)	
H2A	0.5761	−0.1048	0.6120	0.041*	
N3	0.3797 (2)	0.27032 (14)	0.90459 (10)	0.0371 (3)	
N4	0.0141 (2)	0.14250 (14)	0.88879 (10)	0.0358 (3)	
C1	0.2553 (2)	0.45880 (16)	0.67164 (12)	0.0337 (4)	
C2	0.0761 (2)	0.43864 (17)	0.70980 (12)	0.0348 (4)	
C3	−0.0163 (3)	0.5755 (2)	0.62681 (17)	0.0537 (5)	
H3A	−0.1104	0.6137	0.6123	0.064*	
C4	0.1556 (3)	0.5983 (2)	0.59186 (18)	0.0577 (6)	
H4A	0.1817	0.6518	0.5532	0.069*	
C5	0.2918 (3)	0.5393 (2)	0.61535 (15)	0.0484 (5)	
H5A	0.4112	0.5547	0.5924	0.058*	
C6	0.4134 (2)	0.39785 (18)	0.68957 (13)	0.0382 (4)	
C7	0.1537 (2)	−0.09387 (15)	0.58063 (11)	0.0299 (3)	
C8	0.3275 (2)	−0.07335 (15)	0.63130 (11)	0.0296 (3)	
C9	0.4617 (3)	−0.16086 (18)	0.49032 (13)	0.0410 (4)	
H9A	0.5672	−0.1836	0.4614	0.049*	
C10	0.2993 (3)	−0.17670 (18)	0.44038 (13)	0.0426 (4)	
H10A	0.2921	−0.2076	0.3768	0.051*	
C11	0.1431 (3)	−0.14547 (17)	0.48677 (12)	0.0371 (4)	
H11A	0.0285	−0.1597	0.4537	0.045*	
C12	−0.0237 (2)	−0.06449 (17)	0.62609 (12)	0.0348 (4)	
C13	0.5588 (3)	0.3343 (2)	0.91191 (15)	0.0478 (5)	
H13A	0.6175	0.3449	0.8589	0.057*	
C14	0.6625 (3)	0.3863 (2)	0.99610 (18)	0.0597 (6)	
H14A	0.7872	0.4319	0.9990	0.072*	
C15	0.5799 (3)	0.3698 (2)	1.07374 (16)	0.0605 (6)	
H15A	0.6489	0.4033	1.1300	0.073*	
C16	0.3900 (3)	0.30230 (19)	1.06979 (14)	0.0487 (5)	
C17	0.2905 (4)	0.2797 (2)	1.14779 (14)	0.0637 (7)	
H17A	0.3542	0.3082	1.2054	0.076*	
C18	0.1066 (4)	0.2179 (2)	1.13941 (14)	0.0627 (6)	
H18A	0.0455	0.2055	1.1914	0.075*	
C19	0.0049 (3)	0.17163 (19)	1.05273 (13)	0.0457 (5)	
C20	−0.1876 (3)	0.1079 (2)	1.03977 (16)	0.0551 (5)	
H20A	−0.2563	0.0957	1.0898	0.066*	
C21	−0.2720 (3)	0.0644 (2)	0.95414 (16)	0.0555 (5)	
H21A	−0.3993	0.0226	0.9450	0.067*	
C22	−0.1675 (3)	0.0825 (2)	0.87992 (14)	0.0453 (5)	
H22A	−0.2273	0.0512	0.8214	0.054*	
C23	0.2953 (3)	0.25494 (16)	0.98230 (12)	0.0361 (4)	
C24	0.0995 (3)	0.18802 (16)	0.97393 (11)	0.0353 (4)	

### Atomic displacement parameters (Å^2^)

**Table:**

	*U*^11^	*U*^22^	*U*^33^	*U*^12^	*U*^13^	*U*^23^
Mn1	0.02949 (16)	0.04268 (17)	0.03019 (15)	0.00816 (12)	0.00431 (11)	0.01164 (11)
S1	0.0363 (3)	0.0591 (3)	0.0559 (3)	0.0184 (2)	0.0149 (2)	0.0262 (2)
S2	0.0370 (3)	0.0583 (3)	0.0304 (2)	0.0211 (2)	−0.00322 (18)	0.0087 (2)
O1	0.0458 (8)	0.0585 (9)	0.0614 (9)	0.0264 (7)	0.0261 (7)	0.0324 (7)
O2	0.0258 (7)	0.0607 (10)	0.1294 (16)	0.0085 (7)	0.0033 (8)	0.0257 (10)
O3	0.0338 (7)	0.0520 (8)	0.0431 (7)	0.0175 (6)	−0.0025 (6)	0.0015 (6)
O4	0.0251 (7)	0.0556 (9)	0.0845 (11)	0.0056 (6)	0.0012 (7)	0.0055 (8)
N1	0.0258 (8)	0.0488 (9)	0.0600 (10)	0.0101 (7)	0.0018 (7)	0.0155 (8)
N2	0.0237 (7)	0.0384 (8)	0.0398 (8)	0.0068 (6)	0.0034 (6)	0.0088 (6)
N3	0.0309 (8)	0.0392 (8)	0.0397 (8)	0.0069 (6)	0.0023 (6)	0.0091 (6)
N4	0.0312 (8)	0.0431 (8)	0.0337 (7)	0.0068 (6)	0.0039 (6)	0.0126 (6)
C1	0.0252 (8)	0.0355 (9)	0.0390 (9)	0.0051 (7)	0.0017 (7)	0.0093 (7)
C2	0.0256 (8)	0.0366 (9)	0.0396 (9)	0.0069 (7)	−0.0006 (7)	0.0056 (7)
C3	0.0442 (12)	0.0476 (12)	0.0755 (15)	0.0130 (9)	−0.0049 (11)	0.0249 (11)
C4	0.0528 (13)	0.0516 (13)	0.0778 (16)	0.0087 (10)	0.0017 (11)	0.0356 (11)
C5	0.0365 (10)	0.0503 (12)	0.0627 (13)	0.0071 (9)	0.0080 (9)	0.0246 (10)
C6	0.0277 (9)	0.0465 (11)	0.0408 (10)	0.0121 (8)	0.0068 (7)	0.0093 (8)
C7	0.0272 (8)	0.0311 (8)	0.0327 (8)	0.0059 (7)	0.0008 (7)	0.0116 (7)
C8	0.0251 (8)	0.0325 (8)	0.0331 (8)	0.0065 (7)	0.0037 (6)	0.0122 (7)
C9	0.0351 (10)	0.0434 (10)	0.0412 (10)	0.0068 (8)	0.0134 (8)	0.0070 (8)
C10	0.0505 (11)	0.0438 (10)	0.0316 (9)	0.0081 (9)	0.0064 (8)	0.0085 (8)
C11	0.0384 (10)	0.0388 (9)	0.0348 (9)	0.0073 (8)	−0.0041 (8)	0.0121 (7)
C12	0.0268 (9)	0.0444 (10)	0.0365 (9)	0.0113 (8)	−0.0026 (7)	0.0138 (8)
C13	0.0350 (10)	0.0499 (11)	0.0556 (12)	0.0067 (9)	0.0013 (9)	0.0116 (9)
C14	0.0373 (11)	0.0523 (13)	0.0772 (16)	0.0003 (9)	−0.0113 (11)	0.0038 (11)
C15	0.0576 (14)	0.0582 (14)	0.0522 (13)	0.0084 (11)	−0.0176 (11)	−0.0050 (11)
C16	0.0562 (13)	0.0429 (11)	0.0398 (10)	0.0100 (9)	−0.0091 (9)	−0.0005 (8)
C17	0.0877 (19)	0.0659 (15)	0.0296 (10)	0.0140 (14)	−0.0078 (11)	0.0014 (9)
C18	0.0849 (18)	0.0659 (15)	0.0326 (10)	0.0115 (13)	0.0107 (11)	0.0096 (10)
C19	0.0589 (13)	0.0445 (11)	0.0364 (10)	0.0150 (10)	0.0125 (9)	0.0125 (8)
C20	0.0552 (13)	0.0636 (14)	0.0526 (12)	0.0130 (11)	0.0249 (11)	0.0261 (10)
C21	0.0379 (11)	0.0710 (15)	0.0595 (13)	0.0040 (10)	0.0136 (10)	0.0274 (11)
C22	0.0320 (10)	0.0594 (12)	0.0453 (11)	0.0054 (9)	0.0049 (8)	0.0192 (9)
C23	0.0403 (10)	0.0333 (9)	0.0329 (9)	0.0099 (8)	0.0004 (7)	0.0045 (7)
C24	0.0399 (10)	0.0355 (9)	0.0319 (9)	0.0112 (8)	0.0058 (7)	0.0093 (7)

### Geometric parameters (Å, °)

**Table:**

Mn1—O1	2.0868 (14)	C5—H5A	0.9300
Mn1—O3	2.0975 (13)	C7—C11	1.380 (2)
Mn1—N4	2.2627 (15)	C7—C8	1.416 (2)
Mn1—N3	2.2822 (15)	C7—C12	1.514 (2)
Mn1—S1	2.6067 (6)	C9—C10	1.350 (3)
Mn1—S2	2.6347 (5)	C9—H9A	0.9300
S1—C2	1.687 (2)	C10—C11	1.391 (3)
S2—C8	1.7104 (17)	C10—H10A	0.9300
O1—C6	1.254 (2)	C11—H11A	0.9300
O2—C6	1.232 (2)	C13—C14	1.394 (3)
O3—C12	1.253 (2)	C13—H13A	0.9300
O4—C12	1.243 (2)	C14—C15	1.356 (4)
N1—C3	1.345 (3)	C14—H14A	0.9300
N1—C2	1.361 (2)	C15—C16	1.409 (3)
N1—H1A	0.8600	C15—H15A	0.9300
N2—C9	1.350 (2)	C16—C23	1.406 (3)
N2—C8	1.357 (2)	C16—C17	1.433 (3)
N2—H2A	0.8600	C17—C18	1.349 (4)
N3—C13	1.326 (3)	C17—H17A	0.9300
N3—C23	1.359 (2)	C18—C19	1.417 (3)
N4—C22	1.329 (2)	C18—H18A	0.9300
N4—C24	1.349 (2)	C19—C20	1.408 (3)
C1—C5	1.375 (3)	C19—C24	1.409 (3)
C1—C2	1.417 (2)	C20—C21	1.352 (3)
C1—C6	1.506 (2)	C20—H20A	0.9300
C3—C4	1.352 (3)	C21—C22	1.388 (3)
C3—H3A	0.9300	C21—H21A	0.9300
C4—C5	1.387 (3)	C22—H22A	0.9300
C4—H4A	0.9300	C23—C24	1.440 (3)
			
O1—Mn1—O3	108.58 (6)	N2—C8—C7	115.82 (15)
O1—Mn1—N4	157.47 (6)	N2—C8—S2	117.91 (12)
O3—Mn1—N4	89.03 (6)	C7—C8—S2	126.25 (13)
O1—Mn1—N3	92.51 (6)	C10—C9—N2	120.07 (16)
O3—Mn1—N3	157.48 (6)	C10—C9—H9A	120.0
N4—Mn1—N3	72.80 (5)	N2—C9—H9A	120.0
O1—Mn1—S1	84.17 (4)	C9—C10—C11	118.06 (17)
O3—Mn1—S1	93.10 (4)	C9—C10—H10A	121.0
N4—Mn1—S1	80.87 (4)	C11—C10—H10A	121.0
N3—Mn1—S1	96.91 (4)	C7—C11—C10	121.77 (16)
O1—Mn1—S2	96.30 (4)	C7—C11—H11A	119.1
O3—Mn1—S2	83.86 (4)	C10—C11—H11A	119.1
N4—Mn1—S2	99.54 (4)	O4—C12—O3	124.89 (17)
N3—Mn1—S2	86.13 (4)	O4—C12—C7	116.47 (16)
S1—Mn1—S2	176.912 (18)	O3—C12—C7	118.57 (16)
C2—S1—Mn1	107.54 (6)	N3—C13—C14	122.4 (2)
C8—S2—Mn1	100.09 (6)	N3—C13—H13A	118.8
C6—O1—Mn1	138.96 (12)	C14—C13—H13A	118.8
C12—O3—Mn1	135.54 (11)	C15—C14—C13	119.5 (2)
C3—N1—C2	125.06 (17)	C15—C14—H14A	120.3
C3—N1—H1A	117.5	C13—C14—H14A	120.3
C2—N1—H1A	117.5	C14—C15—C16	120.4 (2)
C9—N2—C8	124.92 (15)	C14—C15—H15A	119.8
C9—N2—H2A	117.5	C16—C15—H15A	119.8
C8—N2—H2A	117.5	C23—C16—C15	116.3 (2)
C13—N3—C23	118.40 (17)	C23—C16—C17	119.1 (2)
C13—N3—Mn1	126.38 (14)	C15—C16—C17	124.6 (2)
C23—N3—Mn1	115.22 (12)	C18—C17—C16	121.4 (2)
C22—N4—C24	118.14 (16)	C18—C17—H17A	119.3
C22—N4—Mn1	125.20 (12)	C16—C17—H17A	119.3
C24—N4—Mn1	116.64 (12)	C17—C18—C19	121.1 (2)
C5—C1—C2	119.49 (16)	C17—C18—H18A	119.4
C5—C1—C6	116.59 (16)	C19—C18—H18A	119.4
C2—C1—C6	123.92 (16)	C20—C19—C24	117.10 (18)
N1—C2—C1	115.39 (17)	C20—C19—C18	123.7 (2)
N1—C2—S1	115.66 (14)	C24—C19—C18	119.2 (2)
C1—C2—S1	128.81 (13)	C21—C20—C19	119.64 (19)
N1—C3—C4	120.12 (19)	C21—C20—H20A	120.2
N1—C3—H3A	119.9	C19—C20—H20A	120.2
C4—C3—H3A	119.9	C20—C21—C22	119.6 (2)
C3—C4—C5	118.0 (2)	C20—C21—H21A	120.2
C3—C4—H4A	121.0	C22—C21—H21A	120.2
C5—C4—H4A	121.0	N4—C22—C21	122.91 (19)
C1—C5—C4	121.8 (2)	N4—C22—H22A	118.5
C1—C5—H5A	119.1	C21—C22—H22A	118.5
C4—C5—H5A	119.1	N3—C23—C16	122.99 (18)
O2—C6—O1	125.14 (17)	N3—C23—C24	118.00 (15)
O2—C6—C1	115.33 (18)	C16—C23—C24	119.01 (18)
O1—C6—C1	119.43 (17)	N4—C24—C19	122.58 (18)
C11—C7—C8	119.22 (15)	N4—C24—C23	117.32 (16)
C11—C7—C12	118.48 (14)	C19—C24—C23	120.10 (17)
C8—C7—C12	122.28 (14)		
			
O1—Mn1—S1—C2	17.56 (8)	C9—N2—C8—C7	3.7 (3)
O3—Mn1—S1—C2	−90.82 (8)	C9—N2—C8—S2	−178.04 (14)
N4—Mn1—S1—C2	−179.34 (8)	C11—C7—C8—N2	−2.6 (2)
N3—Mn1—S1—C2	109.40 (8)	C12—C7—C8—N2	175.75 (15)
O1—Mn1—S2—C8	−66.71 (8)	C11—C7—C8—S2	179.31 (13)
O3—Mn1—S2—C8	41.38 (7)	C12—C7—C8—S2	−2.4 (2)
N4—Mn1—S2—C8	129.35 (7)	Mn1—S2—C8—N2	139.27 (12)
N3—Mn1—S2—C8	−158.82 (7)	Mn1—S2—C8—C7	−42.63 (16)
O3—Mn1—O1—C6	110.0 (2)	C8—N2—C9—C10	−1.3 (3)
N4—Mn1—O1—C6	−29.8 (3)	N2—C9—C10—C11	−2.1 (3)
N3—Mn1—O1—C6	−78.0 (2)	C8—C7—C11—C10	−0.7 (3)
S1—Mn1—O1—C6	18.7 (2)	C12—C7—C11—C10	−179.05 (17)
S2—Mn1—O1—C6	−164.4 (2)	C9—C10—C11—C7	3.1 (3)
O1—Mn1—O3—C12	82.40 (19)	Mn1—O3—C12—O4	149.82 (16)
N4—Mn1—O3—C12	−111.92 (19)	Mn1—O3—C12—C7	−33.4 (3)
N3—Mn1—O3—C12	−76.3 (2)	C11—C7—C12—O4	47.6 (2)
S1—Mn1—O3—C12	167.29 (18)	C8—C7—C12—O4	−130.75 (18)
S2—Mn1—O3—C12	−12.21 (18)	C11—C7—C12—O3	−129.52 (18)
O1—Mn1—N3—C13	−16.56 (16)	C8—C7—C12—O3	52.2 (2)
O3—Mn1—N3—C13	143.27 (15)	C23—N3—C13—C14	−0.2 (3)
N4—Mn1—N3—C13	−179.17 (16)	Mn1—N3—C13—C14	−179.96 (15)
S1—Mn1—N3—C13	−100.99 (15)	N3—C13—C14—C15	1.1 (3)
S2—Mn1—N3—C13	79.59 (15)	C13—C14—C15—C16	−0.9 (4)
O1—Mn1—N3—C23	163.68 (12)	C14—C15—C16—C23	−0.1 (3)
O3—Mn1—N3—C23	−36.5 (2)	C14—C15—C16—C17	179.9 (2)
N4—Mn1—N3—C23	1.07 (12)	C23—C16—C17—C18	−2.3 (4)
S1—Mn1—N3—C23	79.25 (12)	C15—C16—C17—C18	177.7 (2)
S2—Mn1—N3—C23	−100.17 (12)	C16—C17—C18—C19	0.7 (4)
O1—Mn1—N4—C22	126.74 (18)	C17—C18—C19—C20	−179.2 (2)
O3—Mn1—N4—C22	−15.55 (16)	C17—C18—C19—C24	1.5 (3)
N3—Mn1—N4—C22	177.95 (17)	C24—C19—C20—C21	0.8 (3)
S1—Mn1—N4—C22	77.74 (15)	C18—C19—C20—C21	−178.5 (2)
S2—Mn1—N4—C22	−99.16 (15)	C19—C20—C21—C22	0.4 (4)
O1—Mn1—N4—C24	−51.5 (2)	C24—N4—C22—C21	−0.3 (3)
O3—Mn1—N4—C24	166.18 (12)	Mn1—N4—C22—C21	−178.51 (16)
N3—Mn1—N4—C24	−0.32 (12)	C20—C21—C22—N4	−0.7 (4)
S1—Mn1—N4—C24	−100.53 (12)	C13—N3—C23—C16	−0.9 (3)
S2—Mn1—N4—C24	82.57 (12)	Mn1—N3—C23—C16	178.93 (14)
C3—N1—C2—C1	−2.9 (3)	C13—N3—C23—C24	178.52 (16)
C3—N1—C2—S1	173.17 (17)	Mn1—N3—C23—C24	−1.7 (2)
C5—C1—C2—N1	3.4 (3)	C15—C16—C23—N3	1.0 (3)
C6—C1—C2—N1	−177.32 (16)	C17—C16—C23—N3	−178.98 (18)
C5—C1—C2—S1	−172.10 (15)	C15—C16—C23—C24	−178.36 (18)
C6—C1—C2—S1	7.2 (3)	C17—C16—C23—C24	1.7 (3)
Mn1—S1—C2—N1	154.39 (12)	C22—N4—C24—C19	1.5 (3)
Mn1—S1—C2—C1	−30.14 (18)	Mn1—N4—C24—C19	179.91 (14)
C2—N1—C3—C4	1.3 (3)	C22—N4—C24—C23	−178.83 (17)
N1—C3—C4—C5	0.0 (4)	Mn1—N4—C24—C23	−0.4 (2)
C2—C1—C5—C4	−2.4 (3)	C20—C19—C24—N4	−1.8 (3)
C6—C1—C5—C4	178.2 (2)	C18—C19—C24—N4	177.53 (18)
C3—C4—C5—C1	0.7 (4)	C20—C19—C24—C23	178.59 (18)
Mn1—O1—C6—O2	135.3 (2)	C18—C19—C24—C23	−2.1 (3)
Mn1—O1—C6—C1	−48.4 (3)	N3—C23—C24—N4	1.4 (2)
C5—C1—C6—O2	30.9 (3)	C16—C23—C24—N4	−179.16 (16)
C2—C1—C6—O2	−148.4 (2)	N3—C23—C24—C19	−178.88 (16)
C5—C1—C6—O1	−145.71 (19)	C16—C23—C24—C19	0.5 (3)
C2—C1—C6—O1	35.0 (3)		

### Hydrogen-bond geometry (Å, °)

**Table:**

*D*—H···*A*	*D*—H	H···*A*	*D*···*A*	*D*—H···*A*
N1—H1A···O2^i^	0.86	1.86	2.654 (2)	152
N2—H2A···O4^ii^	0.86	2.00	2.7476 (19)	145

Symmetry codes: (i) *x*−1, *y*, *z*; (ii) *x*+1, *y*, *z*.
